# On the Anatomy of the Breast

**Published:** 1840-04

**Authors:** 


ART. VIII.—*On the Anatomy of the Breast.* By Sir ASTLEY P. COOPER, Bart., F.R.S., D.C.L., G.C.H., Serjeant-Surgeon to the Queen, &c. &c.—*London*, 1840. 4to, pp. 265. With a volume of Folio Plates.

THIS beautiful and most valuable treatise has reached us at so late a period that we are barely able to announce its publication. We shall give a review of it in our next Number; but we cannot allow the present to appear without bearing to all our readers our earnest exhortation, that they do not wait for our detailed report, but possess themselves of the book itself. It is one which ought to be in the hands of every scientific member of the profession. It is one, moreover, which even the best reviewer can do but imperfect justice to, and which will prove a sure stumbling-block and rock of offence to the superficial scribbler. The text, which contains nothing superfluous, cannot be exhibited, even in fragmentary beauty, on the slender thread of the scissors-critic, "like orient pearls at random strung;" while the plates—those provoking plates—bid defiance to the pen of the most determined excerptor. The plates in the present work, by the way, are singularly fine, and seem to leave nothing to be done by the followers of Sir Astley in the same path. They contain innumerable figures, most carefully drawn, and many of them admirably coloured. There are, in all, twenty-seven plates—viz., fourteen of the female breast, three of the male breast, and ten of the comparative anatomy of the breast.

